# Improving access to the treatment of hepatitis C in low- and middle-income countries: evaluation of a patient assistance programme

**DOI:** 10.1007/s11096-020-01202-1

**Published:** 2020-11-28

**Authors:** Salamat Ali, Tofeeq Ur-Rehman, Mashhood Ali, Sayeed Haque, Faisal Rasheed, Eleri Lougher, Muhammad Sarfraz Nawaz, Vibhu Paudyal

**Affiliations:** 1 Department of Pharmacy, Quaid-i-Azam University, Islamabad, Pakistan; 2 Department of Gastroenterology, Pakistan Institute of Medical Sciences (PIMS), Islamabad, Pakistan; 3 Institute of Clinical Sciences, College of Medical and Dental Sciences, University of Birmingham, Birmingham, UK; 4 UBT Laboratory, Nuclear Medicines, Oncology and Radiotherapy Institute, Islamabad, Pakistan; 5 Abertawe Bro Morgannwg University Health Board, Princess of Wales Hospital, Bridgend, UK; 6 Division of Biologicals, Drug Regulatory Authority of Pakistan, Islamabad, Pakistan; 7 School of Pharmacy, College of Medical and Dental Sciences, University of Birmingham, Birmingham, UK

**Keywords:** Access to drugs, Generic medicines, Hepatitis C treatment, Lower-middle-income country, Patient assistant programme

## Abstract

*Background* Modern antiviral treatments have high cure rates against the hepatitis C virus however, the high cost associated with branded medicines and diagnostic tests, have resulted in poor access for many low-income patients residing in low-and-middle-income countries. *Objective* This study aimed to evaluate the role of a patient assistance programme and generic medicines in improving access to treatment of low-income hepatitis C patients in a low-and-middle-income country. *Setting* A major teaching public hospital in Islamabad, Pakistan. *Methods* Hepatitis C patients who presented and enrolled for the patient assistance programme during 12 months (1st July 2015 and 30th June 2016) were included. Demography, prescription characteristics, the total costs of Hepatitis C treatment, medicine cost supported by the programme, out-of-pocket cost borne by the patient and average cost effectiveness ratio per sustained virologic response were calculated and compared for different generic and branded regimens. *Main outcome measure* cost contribution of patient assistance programme. *Results* A total of 349 patients initiated the treatment through the programme and of those 334 (95.7%) completed the prescribed treatment. There were 294 (88.02%) patients who achieved sustained virologic response. Patient assistance programme contributed medicines cost averaging 60.28–86.26% of the total cost of treatment ($1634.6) per patient. The mean (SE) cost per patient for generic option (Sofosbuvir/Ribavirin) was the lowest [$658.36 (22.3) per patient, average cost effectiveness ratio = $720.1/SVR] than branded option (Sovaldi/Ribavirin) [$2218.66 (37.6) per patient, average cost effectiveness ratio = $2361.8/SVR] of the three available treatment regimens. From patients’ perspectives, the mean (SE) out-of-pocket cost was $296.9 (6.7) which primarily included diagnostic cost (69.9%) of the total cost. *Conclusions* Patient assistance programme, combined with generic brands of newer hepatitis C treatment offered a significant reduction in cost and widens access to hepatitis C treatment in low-and middle-income countries. However, substantial out-of-pocket costs of the treatment presents an important barrier for service access. There is a scope to widen such financial assistance programme to offer other costs attributed to patients, specifically for diagnosis, to widen service use in low-and-middle-income countries.

## Impacts on practice

- Patient Assistance Programme coupled with the use of generic brands of hepatitis C treatments have the potential to widen treatment access in developing countries.
- Such programmes should be extended to widen access to hepatitis C treatments in low-and-middle income countries.

## Introduction

Hepatitis C (HC) is a major liver disease that brings serious health concerns for 71 million infected people worldwide [1–6]. A total of 80% of the global health burden of HC relates to low-and middle-income countries (LMICs) [7]. In 2016, the Global Health Sector Strategy on Viral Hepatitis (GHSS) has been adopted by the World Health Assembly to eradicate hepatitis worldwide with intentions to reduce HC incidence by 80% and mortality by 65% by 2030. The access to the affordable and high quality hepatitis medicines and diagnostics is a key element of this strategy [8].

The socioeconomic status of majority of the patients in LMICs is the main hurdle in accessing newly developed direct acting antivirals (DAAs), which have been proven clinically effective but costly treatment options in high-income countries [9]. A collaborative approach between the government, the service providers and social welfare organizations is obligatory to curtail the high health burden of HC in LMICs [10, 11].

Pakistan harbours a high prevalence (4.6–8%) of HC with approximately 8–10 million infected people and has one of the highest prevalence rates of HC among the LMICs [12, 13]. Pakistan has taken several steps in line with WHO strategy which include registration of DAAs with lower retail prices, voluntary licensing of generic DAAs, inclusion of DAAs in National Essential Drug List (NEDL 2018) and commitment for provision of hepatitis treatment in public hospitals [8, 14]. Pakistan being a LMIC, is spending .9% of gross domestic product (GDP) on health provision [15] and only 1.9% of the population have access to formal insurance product [16].

Taking into account the GHSS for viral hepatitis in LMICs, Patient Assistance Programmes (PAPs) and generic medicines may play a crucial role in supporting the low-income patients who have limited access to hospitals and social security institutions [17]. Various policies and models of PAP have been implemented in different countries worldwide. In Egypt, a national programme was launched which permitted subsidized treatment access and reduced diagnostic costs [18]. Other examples of PAP are Non-Government Organization (NGOs) programmes, state owned programmes and the introduction of co-payment policies by pharmaceutical industries [19–21]. In addition, the LMICs can built substantial dedicated funds through a mixed model encompassing donor and state owned programmes that may support accessing HC treatment in line with GHSS.

In Pakistan, a welfare programme is functional at primary, secondary and tertiary healthcare levels, taking a form of state owned financial PAP, administered through Hospital Social welfare department (SWD). This programme receives funds from donors, Pakistan Bait-ul-Mal and Zakat Council to offer medicines’ for conditions such as HC. Pakistan Bait-ul-Mal (PBM), is an autonomous body established through Act. It is significantly contributing in alleviating poverty through its various services and providing assistance to widows, orphaned and chronically ill patients, as per eligibly criteria approved by Bait-ul-Mal Board. Zakat Councils are responsible for collecting and distributing the Islamic taxes known as *Zakat* and *Ushr* in Pakistan [22].

The practice and policies of such PAP need to be evaluated to identify their role in facilitating the access to HC treatment for socio-economically low-income patients in LMICs, however there is very limited published literature in this area.

### Aim of the study

This study aimed to evaluate the contribution of patient assistance programme (PAP) and the use of generic medicines in improving access to treatment for low-income HC patients in Pakistan.

### Ethics approval

This study is a part of PhD research project of researcher (SA). Ethical approvals were obtained from Bioethical Committee Quaid-i-Azam University Islamabad (DFBS/2015-248), Ethical Review Board Pakistan Institute of Medical Sciences (PIMS) (F.1-1/2015/ERB/SZABMU), Islamabad, and Ethical Committee of Nuclear Medicines, Oncology and Radiotherapy Institute (NORI), Islamabad Pakistan.

## Methods

### Study settings and perspectives

An observational study was undertaken analyzing the data of hepatitis C patients at a gastroenterology department at a large tertiary care hospital (the Pakistan Institute of Medical Sciences, Islamabad).

All HC patients aged 18 years or above with a diagnosed genotype, a quantitative PCR result, who were registered at SWD for the 12 months (1st July 2015 and 30th June 2016) of study period were included in the study. Patients with other types of hepatitis, incomplete diagnosis, incomplete records and those who were not supported through the PAP were excluded.

### Patient entitlement and patient assistance program

**Fig. 1:**
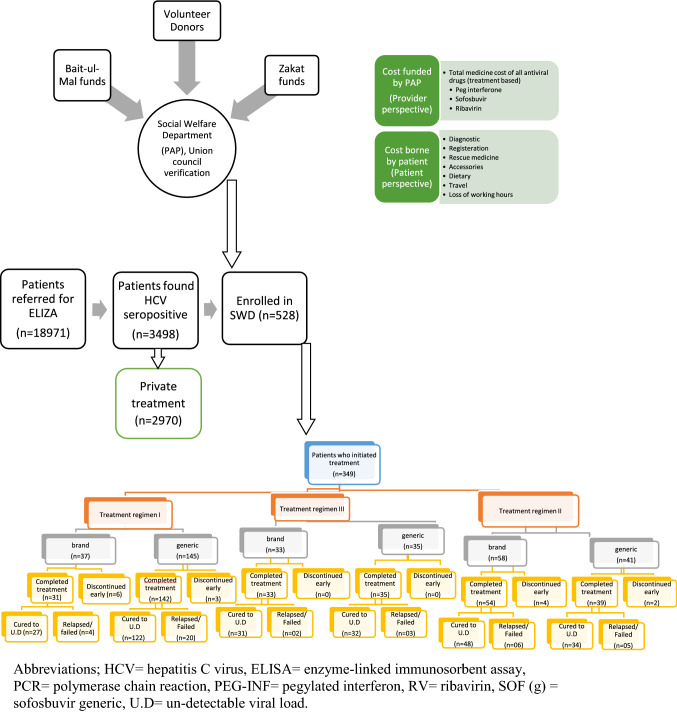
Flow chart of treatment access through social welfare department with a decision tree model showing the treatment choices and outcomes. Abbreviations; *HCV* hepatitis C virus, *ELISA* enzyme-linked immunosorbent assay, *PCR* polymerase chain reaction, *PEG*-*INF* pegylated interferon, *RV* ribavirin, *SOF* (g) sofosbuvir generic, *U.D *= un-detectable viral load

The PAP has a policy to support with the medicines costs for HC patients, after verification of their poor economic status (have assets less than Nisab or monthly income not more than 15,000 PKR = $141.5) [23, 24] from the respective union council and/or local Zakat committee. The HC patients were eligible for entitlement of financial support through Social Welfare Department (SWD) of Pakistan Bait-ul-Mal [25, 26]. The eligible patients are entitled for supply of free medicines, procured through a local contract. All non-medical and indirect costs as out-of-pocket (OOP) expenses are incurred by the patient. PAP financially supported all the registered patients in accessing treatment of HC. This PAP is serving in the real world, as a form of state owned programme that receives funds from Pakistan Bait-ul-Mal to sponsor patients’ medicines (Fig. 1).

### Data sources and collection

Information about demography, diagnosis and treatment were taken from patient medical records available at SWD, responsible for the local administration of PAP. The information about screening and blood tests were collected from the hospital database, Logistics Management Information System (LMIS). Information relating to genotype and PCR (viral load) were collected from a database of Nuclear Medicines, Oncology and Radiotherapy Institute (NORI), Islamabad Pakistan; an associated referral institution for diagnostic tests. All data was anonymized prior to analysis. The cure rate/treatment success was measured as sustained virologic response (SVR). Treatment was considered successful if an un-detectable HCV RNA viral load (VL < 50 IU/mL) was achieved at 24 weeks (SVR24) post end of treatment [27].

### Treatment options

During the study period, both branded medicines for hepatitis C were prescribed as well as generic medicines, which were newly launched in Pakistan. There were six treatment choices; three branded and three generic regimens as followed:

Regimen I [pegylated interferon 180 mcg (brand/generic)/ribavirin (RV)], Regimen II [pegylated interferon 180 mcg/sofosbuvir 400 mg (brand/generic)/RV] and Regimen III [sofosbuvir 400 mg (brand/generic)/RV] (Fig. 1).

### Cost estimates

The treatment costs were based on prescribed treatment regimens according to the individual prescriptions supplied during the study period. The cost of medicines (MC), was taken by multiplying the unit price with the total quantity of medicines for a full course of treatment in accordance with the rates of contract with SWD and the local index of pharmaceuticals, Pharmaguide [28]. Laboratory costs (LC) were calculated in accordance with laboratory rates of PIMS and NORI and included baseline diagnostic and all follow-up tests. The physician charges were calculated as per fee paid by the patient at the registration desk for each hospital visit. To calculate the in-direct costs, the dietary costs incurred (food or drink taken) were calculated based on the government rates contracts approved by PIMS food committee for the fiscal year 2016. Travel expenses were calculated based on the rates specified by Capital Transport Authority taking into account the distance in kilometers between patient’s residence and the hospital [29].

Each patient visit was assumed to experience a loss of eight working hours based on return travel times and length of the consultation inclusive of laboratory testing times. The estimation of indirect cost (IC) was based on a minimum wages per month fixed by the Ministry of Finance, Pakistan in Federal budget 2015–2016 [30]. Unemployed female patients with the status of “house wife’ in the medical records and male patients > 60 years of age were considered “Not earning” and were excluded from the IC analysis [31].

The costs were calculated from a patient’s perspectives, a provider’s perspectives, and societal perspectives.

From patient’s perspectives, out-of-pocket cost (OOP) was calculated which included IC, LC, physician charges, travel fare, dietary expenses, rescue medicines charges (to manage side effects) and cost of consumables (syringes, etc.). From the provider’s perspectives, costs borne by the PAP were calculated. SWD provided all of anti-HC medicines free of charge, which were purchased for HC patients at discounted price (68% of MRP). From a societal perspective, medicine cost savings per individual and total treated patients was recorded.

**Fig. 2:**
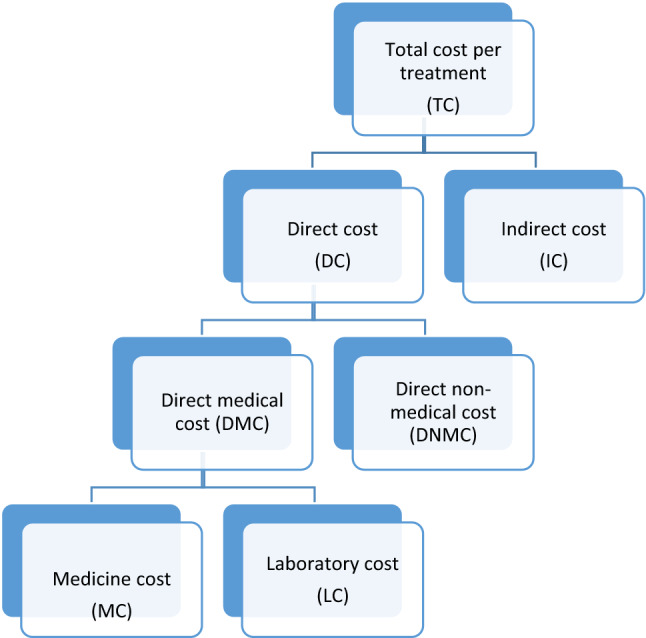
Sub-categories of treatment cost and calculation formulae. *Note*: Formulae used for calculation of costs are: $Toal\,treatment\,cost\,per\,patient = DC + IC$; $Direct\,Cost \left( {DC} \right) = DMC + DNMC$; DMC = MC + LC; $ACER = \frac{{Total \,cost\, of\, a\, treatment\, regimen\, \left( {{\$ }} \right)}}{{number\,of\,patient\,cured\left( {SVR} \right)}}$

Hospital registration charges, medicine cost and laboratory cost was grouped into direct medical cost (DMC) whereas travel costs and dietary expenses were taken as direct non-medical cost (DNMC). The total treatment cost per patient and average cost-effectiveness ratio (ACER) for each treatment regimen was calculated as per equations [32, 33] provided in Fig. 2.

All monetary values were taken initially in Pakistani rupee (PKR) and were adjusted to purchasing power parity (PPP) using the “currency notes exchange rate” in Pakistan on 29.01.2016 (1 USD ($) = 105.95 PKRs), according to National Bank of Pakistan [34].

### Statistical analysis

The demographic variables were summarized using a frequency distribution. The prescription variables (anti HCV treatments and course characteristics) were described using simple descriptive statistics. All costs were calculated as mean ± standard error (SE) and interquartile range (IQR) was calculated where appropriate. Kruskal–Wallis test was performed to determine the differences in the costs of all treatment regimens and Wilcoxon signed-rank test was performed to assess contribution of PAP on treatments costs. The analysis were performed using SPSS software 24.0 for Windows (IBM SPSS Institute, Cary, NC) assisted by a statistician (SH).

## Results

### Enrolment and participant characteristics

During the 12 months of study period, a total of 18,971 patients were referred to the laboratory for screening by ELISA, of which 3498 (18.4%) were found to be HCV seropositive. There were 2970 (84.9%) patients who accessed treatment via private, employer or guardian resources. Only 528 (15.1%) were enrolled in SWD for PAP support and of those, 179 (33.9%) did not progress to commence treatment beyond SWD decision.

There were 349 (66.1%) HC patients who initiated the treatment under PAP support and of those, 334 (95.7%) completed treatment while 15 (4.3%) discontinued treatment early or were lost to follow up. The majority 186 (55.7%) of the patients who completed the treatment were aged between 21 and 40 years. A total of 206 (61.7%) female and 128 (38.3%) male patients completed the prescribed treatment. A total of 204 (61.1%) patients were residents of urban areas and 130 (38.9%) were from rural areas. Ninety eight patients (29.3%) were presented with a high positive viral load and genotype 3a was the most prevalent genotype 324 (97.0%) (Table 1).

**Table 1:** Demographic characteristics of the participants and prescription related parameters

Parameters	Subcategories	Patients who started treatment (n = 349)	Patients who completed treatment (n = 334)	Patients who discontinued treatment (n = 15)
		n(%)	n(%)	n(%)
Age (mean ± SD) Age groups (Years)	01–20 21–40 41–60 61–80 81–100	39.37 ± 10.56 9(2.6) 195(55.9) 139(39.8) 5(1.4) 1(.3)	39.60 ± 10.41 9 (2.7) 186(55.7) 135(40.4) 4(1.2) 0(.0)	39.13 ± 10.70 0(00) 9(60.0) 4(26.6) 1(6.7) 1(6.7)
Gender	Female	216(61.9)	206(61.7)	10(66.67)
	Male	133(38.1)	128(38.3)	05(33.33)
Social Background	Rural	134(38.4)	130(38.9)	04(26.67)
	Urban	215(61.6)	204(61.1)	11(73.33)
Diagnosis	HCV viremic	267(76.5)	261(78.1)	6(40.0)
	Chronic hepatitis	72(20.6)	64(19.2)	8(53.3)
	HCV + Comorbidity	10(2.9)	9(2.7)	1(6.7)
Genotype	1b	5(1.4)	5(1.5)	0(00)
	2b	2(.6)	2(.6)	0(00)
	3a	339(97.1)	324(97.0)	15(100)
	Un-type able	3(.9)	3(.9)	0(00)
Viral load (before Tx)	Very low + ve	7(2.0)	7(2.1)	0(00)
	Low +ve	114(32.7)	107(32.0)	7(46.7)
	+ve	123(35.2)	122(36.5)	1(6.6)
	High + ve	105(30.1)	98(29.3)	7(46.7)

Viral load is represented as; Very low +ve = Less than 8000 IU/ml, Low +ve = 8001–20,000 IU/ml, +ve = 20,001-800,000 IU/ml, and High +ve = Greater than 800,000 IU/ml, HCV = HC virus

*HCV* HC virus, *Tx* treatment

### Treatment regimens and costs-effectiveness

A total of 173(51.8%) patients were treated with regimen I [(branded, n = 31), (generic, n = 142)], 93(27.8%) were treated with regimen II [(branded, n = 54), (generic, n = 39)] and 68(20.4%) with regimen III [(branded, n = 33), (generic, n = 35)] (Table 2 and Fig. 1).

**Table 2:** Cost characteristics and Average Cost Effectiveness Ratio (ACER) of branded and generic anti-HC treatment regimens

Treatment combinations (n = 334)	Total cost of treatment (all costs are in USD)				Treatment outcomes		Average cost effectiveness ratio (ACER)	Kruskal–Wallis test
	Total cost of all patients	Mean(SE)	Minimum	Maximum	Relapsed/Failed n(%)	SVR24 n(%)	$/SVR24	Mean rank
Regimen I (b) (n = 31)	73,473.36	2370.11(23.24)	2216.32	2829.22	4(12.9)	27(87.1)	2721.22	313.03
Regimen I (g) (n = 142)	225,169.36	1585.70(18.78)	1332.14	2244.13	20(14.1)	122(85.9)	1845.65	154.89
Regimen II (b) (n = 54)	101,519.30	1879.99(29.57)	1698.28	2428.55	6(11.1)	48(88.9)	2114.98	217.34
Regimen II (g) (n = 39)	49,538.32	1270.21(27.55)	897.73	1504.83	5(12.8)	34(87.2)	1457.01	67.85
Regimen III (b) (n = 33)	73,215.95	2218.66(37.55)	1242.60	2766.19	2(6.1)	31(93.9)	2361.80	279.64
Regimen III (g) (n = 35)	23,042.47	658.36(22.30)	420.46	980.68	3(8.6)	32(91.4)	720.08	18.17

SVR24 = Sustained virological response at 24 weeks post end of treatment; Viral load based on sensitivity of PCR equipment (Rotor gene real-time PCR system); if it is less than 50 IU/ml = Un-detectable

^Kruskal–Wallis test shows mean rank of treatment combinations and the combination with the least rank (SOF generic) is adaptable^

*HC* hepatitis C, *b* = brand, *g* = generic, *SE* standard error, *ACER* average cost effectiveness ratio, *USD* U.S dollar

Overall, a total of 294(88.02%) out of 334 patients achieved a SVR24. Table 2 shows the comparison between groups for treatment costs. The lowest ACER was $720.08/SVR24 for generic regimen III and the highest ACER was $2721.2/SVR24 for branded regimen III. The mean costs of all treatment regimens were significantly different as revealed by Kruskal–Wallis test (X^2^ = 121.54, df = 5 and *p* value = .000) (Table 2).

The total cost for treating the whole cohort of 334 patients was $545,958.76 and the mean (SE) cost per treatment was $1634.6(27.4). The total cost of treating 142 patients with 24 weeks course of regimen I (generic) was $225,169.36 while the total cost for treating 54 patients with 12 weeks course of regimen II (branded) was $101,519.30. The mean (SE) cost was $2218.66 (37.55) per patient per treatment of regimen III (branded) while the mean (SE) was $658.36(22.30) per patient per treatment with regimen III (generic) (Table 2).

**Fig. 3:**
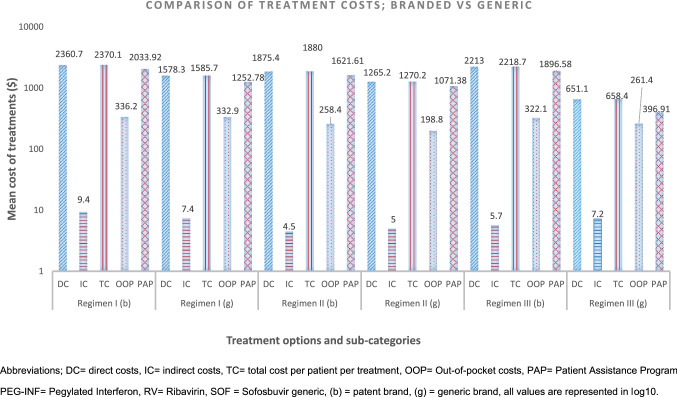
Comparison of treatment regimens based on direct and indirect costs. Abbreviations; *DC* direct costs, *IC* indirect costs, *TC* total cost per patient per treatment, *OOP* Out-of-pocket costs, PAP = Patient Assistance Program, *PEG*-*INF* Pegylated Interferon, RV = Ribavirin, *SOF* Sofosbuvir generic, (b) = patent brand, (g) = generic brand, all values are represented in log10

In all six treatment options, direct medical costs were higher than the direct non-medical (primarily the cost of diagnostic tests) and indirect costs (Fig. 1). The cost of the medicines (MC) was fully supported by PAP. The most expensive MC was $1896.58(27.8) for regimen III (branded) and the least expensive MC was $396.91(16.0) for regimen III (generic). Figure 3 shows the details of sub-categories of treatment costs (TC).

The mean (SE) OOP costs for all patients (n = 334) were $296.9(6.72). Comparing all regimens, the mean (SE) OOP costs were [$336.18(23.99), $332.92(11.44)], [$258.38(7.80), $198.38(10.63)] and [$322.08(22.08), $261.45(14.78)] for regimen I (branded, generic), regimen II (branded, generic) and regimen III (branded, generic) respectively. The least OOP expense of $198.83(10.63) was attributed to the regimen II (generic) for a 12 weeks treatment course (Table 3).

**Table 3:** Comparison of treatment cost per patient, PAP contribution and Out-of-Pocket costs between branded vs generic anti-HC regimens

Cost sub-categories (all costs are in USD)	All treatment (n = 334)	Treatment Regimen I				Treatment Regimen II				Treatment Regimen III			
		(Brand) (n = 31)		(Generic) (n = 142)		(Brand) (n = 54)		(Generic) (n = 39)		(Brand) (n = 33)		(Generic) (n = 35)	
	Total, Mean(SE)	Mean	(SE)	Mean	(SE)	Mean	(SE)	Mean	(SE)	Mean	(SE)	Mean	(SE)
Pegylated interferon	291,529.2, 872.84(31.18)	1925.44	(.00)	1145.16	(16.67)	661.49	(27.70)	859.16	(29.02)	N/A	(N/A)	N/A	(N/A)
Ribavirin	30,887.5, 92.48(1.71)	108.49	(3.40)	107.61	(.68)	54.71	(1.20)	46.07	(3.99)	113.21	(2.03)	107.33	(6.53)
Sofosbuvir	124,358.8, 372.33(31.15)	N/A	(N/A)	N/A	(N/A)	905.41	(.00)	166.15	(.00)	1783.38	(27.44)	289.58	(12.45)
MC = Cost spent by PAP	446,775.56, 1337.65(25.88)	2033.92	(3.40)	1252.78	(16.75)	1621.61	(27.44)	1071.38	(27.72)	1896.58	(27.83)	396.91	(16.01)
*Diagnostic and follow*-*up laboratory costs (OOP*-*1)*													
Blood/serological tests	5384.3, 16.12(.23)	18.68	(.35)	18.18	(.23)	10.74	(.33)	11.04	(.29)	17.46	(.68)	18.21	(.32)
Genotyping test	23,643.2, 70.79(.0)	70.79	(.00)	70.79	(.00)	70.79	(.00)	70.79	(.00)	70.79	(.00)	70.79	(.00)
PCR test	40,302.0, 120.66(1.68)	130.01	(2.13)	129.81	(1.02)	132.14	(.00)	74.54	(5.52)	144.15	(6.07)	86.83	(5.92)
Total OOP-1	69,329.5, 207.6(1.75)	219.47	(2.13)	218.78	(1.11)	213.67	(.33)	156.36	(5.56)	232.39	(6.25)	175.83	(6.03)
*Other OOP costs (OOP*-*2)*													
Rescue medicines (to manage side effects)	75.5, .23(.08)	.00	(.00)	.04	(.02)	.27	(.12)	.95	(.17)	00	(.00)	.50	(.19)
Accessories (Syringes)	403.2, 1.21(.03)	1.81	(.00)	1.81	(.00)	.91	(.00)	.91	(.00)	.05	(.01)	.10	(.07)
Physician charges	77.6, .23(.00)	.27	(.01)	.27	(.00)	.14	(.00)	.14	(.00)	.25	(.01)	.27	(.00)
Dietary expenses	1683.0, 5.0(.08)	6.03	(.07)	5.93	(.06)	3.10	(.02)	3.11	(.00)	5.44	(.24)	5.31	(.23)
Travelling costs	25,389.4, 76.0(6.39)	99.23	(23.77)	98.69	(11.27)	35.76	(7.64)	32.32	(9.86)	78.23	(14.03)	72.19	(9.20)
Indirect costs	2224.9, 6.7(.54)	9.37	(2.05)	7.40	(.92)	4.54	(.81)	5.03	(.98)	5.70	(1.72)	7.24	(1.87)
Total OOP-2	29,853.66, 89.38(6.43)	116.71	(23.64)	114.14	(11.25)	44.71	(7.77)	42.47	(9.74)	89.67	(24.23)	85.62	(15.91)
Total OOP Costs (1 + 2) (Patient’s perspective costs)	99,183.2, 296.95(6.72)	336.18	(23.99)	332.92	(11.44)	258.38	(7.80)	198.83	(10.63)	322.08	(22.08)	261.45	(14.78)
Cost per patient *p*-value (Wilcoxon SR)*	545,958.76, 1634.61(27.39)	2370.11 *p *= .000	(23.24.)	1585.70 *p *= .000	(18.78)	1879.99 *p *= .000	(29.57)	1270.21 *p *= .000	(27.55)	2218.66 *p *= .000	(37.55)	658.36 *p *= .000	(22.30)
Cost saved from societal perspective by rebates prices	42,968.18	20,176.49	56,926.32	28,021.42	13,370.82	20,027.88	4445.39						

*HC* hepatitis C, *USD *= U.S dollar, *SE* standard error, *MC *= medicine cost, *PCR *= polymerase chain reaction, *OOP* out-of-pocket, *PAP* patient assistant program

*Wilcoxson signed ranks test; a *p*-value < .05 was considered significant

The PAP scheme, significantly contributed of the total cost per patient as revealed by Wilcoxon signed-rank test (W-SR), (Z = − 4.860, *p *= .000) for regimen I (branded) vs regimen I (generic) sponsoring $2033.92 out of $2370.11. Similarly, the regimen III showed a W-SR test value; Z = − 4.979, *p *= .000 for total cost per patient vs PAP cost. Detailed comparison is given in Table 3.

## Discussion

This study has evaluated the treatment costs, the contribution of generic medicines and role of a Patient Assistance Programme (PAP) in improving access to treatment of HC in Pakistan. The results showed that the PAP has supported medicine cost ranging from 60.28 to 86.26% of the total cost of treatment depending upon regimen type. The least expensive regimens was SOF/RV (generic) costing $658.36(22.30) per patient while the most expensive regimenwas branded Sovaldi/RV with a mean (SE) cost of $2218.66(37.55) per patient. The treatment outcomes of generic sofosbuvir in terms of SVR24 were comparable to branded regimens as it cured 91.4% of the patients’ vs branded Sovaldi (93.9%). These cure rate findings are conformational to recent studies for the newer DAA based antiviral regimens [35, 36].

Our estimate showed that the treatment cost was significantly less for generic options particularly regimens containing DAAs (regimen II and III). PAP further saved the cost of medicines via the contract purchase on discounted price. WHO, and numerous public sector organizations have successfully implemented the cost effective bulk purchase/contract strategy, to provide treatment options for the maximum number of patients, within the limited resources available [37–39].

Currently, the success of DAA based anti HC therapies has outclassed the interferon based treatment regimens associated with low cure rate and greater side effects [40]. In this study, treatment outcomes of both the DAA based treatment regimens are comparable (Table 2). The selection of generic DAAs for all of HC patients may save an average of $984.8 per patients, resulting in the ability for SWD to treat an additional 500–600 HC patients.

Although PAP, with the selection of generic HC medicine and contractual supplies, has supported low-income HC patients accessing HC treatments, the patients still incurred laboratory cost, direct non-medical cost, and indirect cost ($296.9 per patients) which accounts from 13 to 39% of the total treatment cost. Such OOP costs despite being significantly lower as compared to the medicine costs paid by the PAP, are often unaffordable for the poorest of the population with $1641 Gross Domestic Product (GDP) per capita (nominal) [41]. Resultantly, there were considerable numbers of patients who did not commenced treatment beyond SWD decision, and some registered patients discontinued the treatment, or were lost to follow-up during post-medication phase and thus did not obtain a confirmed SVR24 (Fig. 1).

Looking into the details of OOP from the patients’ perspectives, it appears that laboratory cost amounts to be 64.9%, 65.7%, 82.7%, 78.6%,72.2% and 67.2% of total OOP costs for treatments options I–VI respectively. WHO has recommended the contract laboratory services for economical and quality tests [42, 43]. Health managers and policy makers of PAP needs to review the WHO strategy for the benefit of the non-affording patients. The allocation of funds for support of low-cost laboratory tests at contractual discounted prices may enhance the access to HC treatments.

This study has undertaken the economic and clinical evaluation of the number of HC patients accessing treatment through PAP which is novel in Pakistan. Collecting data from a large hospital and allied institutes with an adequate patient sample size strengthen this study as did the use of validated and recognised data sources for all information on costs and clinical outcomes.

This study however has a number of limitations. Firstly, this study has a fair percentage of patients who discontinued treatment early or were lost to follow up who may/may not have achieved the positive treatment outcomes. Secondly, the study protocol would have been strengthened with identification of relapsed cases. In addition, due to the late registration of DAAs in Pakistan, the number of patients receiving sofosbuvir containing regimen was small. The study was unable to calculate the costs from a caregiver perspective and cost of the treatment for self-financed patients.

Given the high prevalence of HC in Pakistan, there is a need for a collaborative effort between WHO, National Hepatitis Control Program, SWD, hospital administration and other NGOs to allocate more funds for low-cost generic medicines and laboratory test so that the target of eradicating HC by 2030 may be achieved in LMICs [44]. Patient financial support programmes similar to PAP are practicable for other LMICs for the welfare of general public. This effort will add to the strong suit of health system to combat HC. A future study exploring the similar economic evaluation of PAP at multiple tertiary and secondary care hospitals will strengthen the above recommendations.

Hospital clinical and community pharmacists have demonstrated positive contribution to medicines adherence and clinical outcomes in HC patients [45, 46]. Improving these outcomes can minimise OOP for patients reducing the number of visits to clinic and preventing treatment failures [47].

## Conclusions

PAP, combined with generic brands of newer HC treatment offered a significant reduction in cost and widens access to HC treatment in Pakistan. However, substantial OOP costs of the treatment presents an important barrier for service access. There is a scope to widen such financial assistance programs to offer other costs attributed to patients to widen service use in LMICs. The national and global health mangers need to investigate economical solutions for support of low-cost laboratory tests. Future research work to explore the rationale of such PAP at multiple centers and in other LMICs will strengthen these findings.

## Data statement section

The original files, datasets used and analysed in the current study are available from the custodian of the data: tofeeq.urrehman@qau.edu.pk.

## References

[1] World Health Organization (a) (WHO). Fact sheets/Detail/HC. https://www.who.int/en/news-room/fact-sheets/detail/hepatitis-c (2018). Accessed 12 Dec 2018.

[2] Alavian S-M. HC virus infection: epidemiology, risk factors and prevention strategies in public health in IR IRAN. Gastroenterol Hepatol Bed Bench. 2009;3(1).

[3] Shepard CW, Finelli L, Alter MJ (2005). Global epidemiology of HC virus infection. Lancet Infect Dis.

[4] Iyengar S, Tay-Teo K, Vogler S, Beyer P, Wiktor S, de Joncheere K (2016). Prices, costs, and affordability of new medicines for HC in 30 countries: an economic analysis. PLoS Med.

[5] Roux P, Sagaon-Teyssier L, Lions C, Fugon L, Verger P, Carrieri M (2014). HCV seropositivity in inmates and in the general population: an averaging approach to establish priority prevention interventions. BMJ Open.

[6] Lozano R, Naghavi M, Foreman K, Lim S, Shibuya K, Aboyans V (2012). Global and regional mortality from 235 causes of death for 20 age groups in 1990 and 2010: a systematic analysis for the global burden of disease study 2010. Lancet.

[7] Mohd Hanafiah K, Groeger J, Flaxman AD, Wiersma ST (2013). Global epidemiology of HC virus infection: new estimates of age-specific antibody to HCV seroprevalence. Hepatology.

[8] World Health Organization (b). Global report on access to HC treatment. Focus on overcoming barriers. http://apps.who.int/iris/bitstream/handle/10665/250625/WHO-HIV-2016.20-eng.pdf?sequence=1 (2016). Accessed 13 Oct 2018.

[9] Bansal S, Singal AK, McGuire BM, Anand BS (2015). Impact of all oral anti-HC virus therapy: a meta-analysis. World J Hepatol.

[10] Hill A, Cooke G (2014). HC can be cured globally, but at what cost?. Science.

[11] Cooke GS (2017). Scaling-up HCV treatment to achieve WHO targets by 2030. Trop Med Int Health.

[12] Umer M, Iqbal M (2016). HC virus prevalence and genotype distribution in Pakistan: comprehensive review of recent data. World J Gastroenterol.

[13] Raja NS, Janjua KA (2008). Epidemiology of HC virus infection in Pakistan. J Micro Immun Infect.

[14] Graham CS, Swan T (2015). A path to eradication of HC in low-and middle-income countries. Antivir Res.

[15] Nishtar S, Bhutta ZA, Jafar TH, Ghaffar A, Akhtar T, Bengali K (2013). Health reform in Pakistan: a call to action. Lancet.

[16] Chaudhry TT, Nabeel F. Microinsurance in Pakistan: progress, problems, and prospects. Lahore J Ecol 2014;18(SE):335–374.

[17] Zullig LL, Wolf S, Vlastelica L, Shankaran V, Zafar SY (2017). The role of patient financial assistance programs in reducing costs for cancer patients. J Manag Care Spec Pharm.

[18] Kaplan K. Low-and middle-income countries defuse HC. HIV Treat Bull. http://i-base.info/htb/21506 (2013). Accessed 15 June 2018.

[19] Younossi Z, Park H, Saab S, Ahmed A, Dieterich D, Gordon S (2015). Cost-effectiveness of all-oral ledipasvir/sofosbuvir regimens in patients with chronic HC virus genotype 1 infection. Aliment Pharmacol Ther.

[20] Ahmadiani S, Nikfar S (2016). Challenges of access to medicine and the responsibility of pharmaceutical companies: a legal perspective. DARU J Pharm Sci.

[21] Stadhouders N, Kruse F, Tanke M, Koolman X, Jeurissen P. Effective healthcare cost-containment policies: a systematic review. Health Policy. 2018. 10.1016/j.healthpol.2018.10.015 30429060

[22] Khan MR, Ali U (2009). An analysis of individual finance assistance program under the Pakistan Bait-ul-Maal. Stat Sci.

[23] Pranam D (2013). Zakat as a measure of social justice in Islamic finance: an accountant’s overview. J Emerg Econ Islam Res.

[24] Sayeed A, editor. Social protection in Pakistan: concept, situation analysis and the way forward. Proceedings of a Joint Seminar Organized by the Planning Commission, ILO and UNDP on Employment-based Poverty Reduction Strategy for Decent Work in Pakistan Pakistan Institute of Development Economics Islamabad. http://researchcollective.org/Documents/Social_Protection_Way_Forward.pdf (2004). Accessed 14 Dec 2018.

[25] Ali M, Rafi S (2013). Medical social work in Pakistan: a multi-model approach to collaborative practice in health care settings. Acad Res Int.

[26] Maal PBu. Criteria for HC patients 2017. http://www.pbm.gov.pk/forms.html (2017). Accessed 10 Aug 2017.

[27] Liver EAfTSoT. EASL recommendations on treatment of HC 2016. J Hepatol. 2017;66(1):153. 10.1016/j.jhep.2016.09.001 27667367

[28] Pharmaguide. 24 ed: Pharmaguide publishing company, Paramount; 2016. https://www.epharmaguide.com/ (2016). Accessed 20 June 2016.

[29] Regional Transport Authority I. public transport; fare 2016. https://ictadministration.gov.pk/ (2016). Accessed 20 June 2016.

[30] Division F. Annual budget statement. In: Division F, editor. Federal budget 2015–2016. http://www.finance.gov.pk/budget/abs_2015_16.pdf (2018). Accessed 20 June 2018.

[31] Zare F, Fattahi MR, Sepehrimanesh M, Safarpour AR. Economic burden of HC virus infection in different stages of disease: a report from Southern Iran. Hepat Mon. 2016;16(4). 10.5812/hepatmon.32654 PMC4887962 27257424

[32] Trask LS, Chapter L. Pharmacoeconomics: principles, methods, and applications. In: Pharmacotherapy: a pathophysiologic approach 8th ed. New York: McGraw-Hill Global Education Holdings. http://accesspharmacy.mhmedical.com/content.aspx (2011). Accessed 2 Dec 2018.

[33] Arnold RJ (2016). Pharmacoeconomics: from theory to practice.

[34] Pakistan NBoP. Rate Sheet-NBP-National Bank of Pakistan 2016 [updated 29.01.2016. https://www.nbp.com.pk/RateSheet/index.aspx (2016). Accessed 11 June 2018.

[35] Pockros PJ, Reddy KR, Mantry PS, Cohen E, Bennett M, Sulkowski MS (2016). Efficacy of direct-acting antiviral combination for patients with HC virus genotype 1 infection and severe renal impairment or end-stage renal disease. Gastroenterology.

[36] Martin NK, Vickerman P, Dore GJ, Grebely J, Miners A, Cairns J (2016). Prioritization of HCV treatment in the direct-acting antiviral era: an economic evaluation. J Hepatol.

[37] Reich MR. Public-private partnerships for public health. Publ Priv Partnersh Publ Health. 2002;1–18.

[38] World Health Organization (c). Global tuberculosis control: epidemiology, strategy, financing: WHO report 2009. https://www.google.com/search?q=World+Health+Org.+2009.+Global+tuberculosis+control%3A+epidemiology%2C+strategy%2C+financing%3A+WHO+report+2009.+&ie=utf-8&oe=utf-8&client=firefox-b (2009). Accessed 20 Dec 2018.

[39] World Health Organization (d). Important new agreement on HC treatment signed. https://medicinespatentpool.org/mpp-media-post/the-medicines-patent-pool-signs-licence-with-abbvie-to-expand-access-to-key-hepatitis-c-treatment-glecaprevirpibrentasvir/ (2018). Accessed 20 Dec 2018.

[40] Asselah T, Boyer N, Saadoun D, Martinot-Peignoux M, Marcellin P (2016). Direct-acting antivirals for the treatment of HC virus infection: optimizing current IFN-free treatment and future perspectives. Liver Int.

[41] Wikipedia. Economy of Pakistan. https://en.wikipedia.org/wiki/Economy_of_Pakistan (2018). Accessed 16 Dec 2018.

[42] Ti L, Kaplan K, Hayashi K, Suwannawong P, Wood E, Kerr T (2013). Low rates of HC testing among people who inject drugs in Thailand: implications for peer-based interventions. J Pub Health.

[43] World Health Organization (e). WHO urges countries to scale up HC treatment 2018. https://www.who.int/hepatitis/news-events/hep-c-access-report-2018-story/en/ (2018). Accessed 12 Dec 2018.

[44] Hellard M, Sacks-Davis R, Doyle J (2016). HC elimination by 2030 through treatment and prevention: think global, act in local networks. J Epidemiol Commun Health.

[45] Ali S, Ur-Rehman T, Lougher E, Mutimer D, Ali M, Paudyal V (2020). Impact of HIV and chronic kidney disease comorbidities on HC treatment choices, drug-drug interactions and HC cure. Int J Clin Pharm.

[46] Ali S, Ali M, Paudyal V, Rasheed F, Ullah S, Haque S, Ur-Rehman T (2019). A randomized controlled trial to assess the impact of clinical pharmacy interventions on treatment outcomes, health related quality of life and medication adherence among HC patients. Patient Pref Adher.

[47] Bowen M, Marwick S, Marshall T, Saunders K, Burwood S, Yahyouche A (2019). Multi-morbidity and emergency department visits by a homeless population: a database study in specialist general practice. Br J Gen Pract.

